# High Capability of Pentagalloylglucose (PGG) in Inhibiting Multiple Types of Membrane Ionic Currents

**DOI:** 10.3390/ijms21249369

**Published:** 2020-12-09

**Authors:** Wei-Ting Chang, Ping-Yen Liu, Sheng-Nan Wu

**Affiliations:** 1 Institute of Clinical Medicine, College of Medicine, National Cheng Kung University, Tainan 70101, Taiwan; cmcvecho2@gmail.com (W.-T.C.); larry@mail.ncku.edu.tw (P.-Y.L.); 2 Division of Cardiovascular Medicine, Chi-Mei Medical Center, Tainan 71004, Taiwan; 3 Department of Biotechnology, Southern Taiwan University of Science and Technology, Tainan 71004, Taiwan; 4 Division of Cardiology, Department of Internal Medicine, National Cheng Kung University Hospital, College of Medicine, National Cheng Kung University, Tainan 70401, Taiwan; 5 Department of Physiology, National Cheng Kung University Medical College, No. 1, University Road, Tainan 70101, Taiwan; 6 Institute of Basic Medical Sciences, National Cheng Kung University Medical College, Tainan 70101, Taiwan; 7 Department of Medical Research, China Medical University Hospital, China Medical University, Taichung 40402, Taiwan

**Keywords:** pentagalloylglucose (penta-*O*-galloyl-β-d-glucose), delayed-rectifier K^+^ current, *erg*-mediated K^+^ current, proton-activated Cl^−^ current, hyperpolarization-activated cation current, voltage-gated Na^+^ current, pituitary cell, heart cell

## Abstract

Pentagalloyglucose (PGG, penta-*O*-galloyl-β-d-glucose; 1,2,3,4,6-pentagalloyl glucose), a pentagallic acid ester of glucose, is recognized to possess anti-bacterial, anti-oxidative and anti-neoplastic activities. However, to what extent PGG or other polyphenolic compounds can perturb the magnitude and/or gating of different types of plasmalemmal ionic currents remains largely uncertain. In pituitary tumor (GH_3_) cells, we found out that PGG was effective at suppressing the density of delayed-rectifier K^+^ current (*I*_K(DR)_) concentration-dependently. The addition of PGG could suppress the density of proton-activated Cl^−^ current (*I*_PAC_) observed in GH_3_ cells. The IC_50_ value required for the inhibitory action of PGG on *I*_K(DR)_ or *I*_PAC_ observed in GH_3_ cells was estimated to be 3.6 or 12.2 μM, respectively, while PGG (10 μM) mildly inhibited the density of the *erg*-mediated K^+^ current or voltage-gated Na^+^ current. The presence of neither chlorotoxin, hesperetin, kaempferol, morin nor iberiotoxin had any effects on *I*_PAC_ density, whereas hydroxychloroquine or 4-[(2-butyl-6,7-dichloro-2-cyclopentyl-2,3-dihydro-1-oxo-1H-inden-5yl)oxy] butanoic acid suppressed current density effectively. The application of PGG also led to a decrease in the area of voltage-dependent hysteresis of *I*_PAC_ elicited by long-lasting isosceles-triangular ramp voltage command, suggesting that hysteretic strength was lessened in its presence. In human cardiac myocytes, the exposure to PGG also resulted in a reduction of ramp-induced *I*_K(DR)_ density. Taken literally, PGG-perturbed adjustment of ionic currents could be direct and appears to be independent of its anti-oxidative property.

## 1. Introduction

Pentagalloylglucose (PGG, penta-*O*-galloyl-β-d-glucose, 1,2,3,4,6-pentagalloyl glucose) is a highly bioavailable polyphenolic compound, which is one of the most potent antioxidants in the tannins group (i.e., a gallotannin and the precursor of ellagitannins). This compound has been widely recognized to possess antimicrobial, anti-viral (including in the treatment with *coronavirus* disease [COVID-19]), anti-diabetic, anti-inflammatory and anti-tumor properties [1,2,3,4,5,6,7,8,9]. For example, tannins and related compounds were previously demonstrated to suppress the replication of human immunodeficiency viruses [1]. By using a lentivirus-based pseudotyping system for human respiratory syncytial viruses, recent studies have also disclosed the ability of PGG to inhibit the entry of viral particles [10]. Moreover, tannic acid, a higher galloylated PGG, is a specific form of tannin which has been previously tailored to capture and quantify the exocytotic event in endocrine and neuroendocrine cells [11,12]. PGG were reported to antagonize insulin receptor/insulin-like growth factor-1 receptor (IR/IGF1R), hence sabotaging pancreatic cancer cells and ameliorating cancer cachexia [4,9]. Additionally, this compound was noticed to help stabilize the elastin and collagen in vascular tissues and to restore the biomechanical properties of the arterial extracellular matrix [13,14,15].

The voltage-gated K^+^ (K_V_) channels are crucial in determining membrane excitability in electrically excitable or even non-excitable cells [16,17,18]. Noticeably, K_V_3 (*KCNC*) and K_V_2 (*KCNB*) channels constitute two types of delayed-rectifier K^+^ currents (*I*_K(DR)_), and they are widespread in different excitable cells that include endocrine cells [18,19,20,21]. The biophysical properties of K_V_3.1-K_V_3.2 channels, which are the dominant factors of *I*_K(DR)_ identified in pituitary tumor (GH_3_) cells, are characterized by a positively shifted voltage dependency as well as by fast deactivation rate of the current [19,20,22]. Earlier studies have demonstrated that biologically active flavonoids, which are polyphenols (e.g., hesperetin, kaempferol and morin), can regulate the activity of K_V_ channels [23,24].

There is growing evidence to disclose the functional expression of proton-activated Cl^−^ channels (PAC) in different cell types, although PAC channels tend to share no sequence similarity to any other known ion channels [25,26]. Overexpression of human PAC cDNA in *PAC* knockout cells was observed to generate large proton-activated Cl^−^ currents [26]. The PAC channels were also noticed to constitute whole-cell proton-activated Cl^−^ current (*I*_PAC_), which is biophysically characterized by a strong outwardly rectifying current–voltage relationship [25,26]. The magnitude of PAC-channel activity was revealed to be intimately connected through acid-induced neuronal cell death; consequently, it could be partially protected from ischemic brain injury [26,27]. However, whether and how the presence of PGG or other related compounds is able to adjust the strength and kinetic gating of above-stated types of *I*_K(DR)_ or *I*_PAC_ still remain largely uncertain.

In light of the initiatives stated above, the aim of this work is to explore whether PGG and the related compounds are capable of interacting with plasmalemmal ion channels to perturb the amplitude and gating of membrane ionic currents. The ionic currents studied comprise different types of ionic currents, namely, delayed-rectifier K^+^ current (*I*_K(DR))_, *erg*-mediated K^+^ current (*I*_K(erg)_), proton-activated Cl^−^ current (*I*_PAC_), hyperpolarization-activated cation current (*I*_h_) and voltage-gated Na^+^ current (*I*_Na_) in a well-characterized electrically excitable cell model (e.g., pituitary tumor [GH_3_] cells). Moreover, its effect on the density of ramp-induced *I*_K(DR)_ in human cardiac myocytes (HCM) was evaluated in this study.

## 2. Results

### 2.1. Effect of PGG on Delayed-Rectifier K^+^ Current (I_K(DR)_) Recorded from Pituitary Tumor (GH_3_) Cells

**Figure 1:**
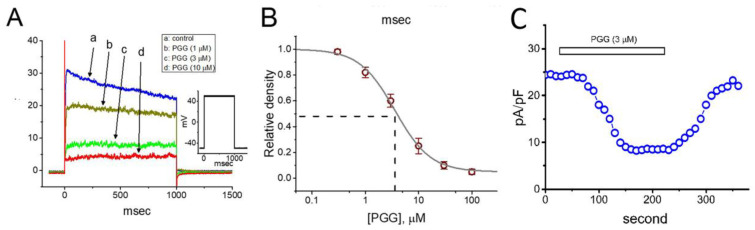
Inhibitory effect of PGG on the amplitude of delayed-rectifier K^+^ current (*I*_K(DR)_) measured from pituitary tumor (GH_3_) cells. In these whole-cell experiments, we kept cells to be bathed in Ca^2+^-free Tyrode’s solution containing 1 μM tetrodotoxin, and the recording pipette was filled with K^+^-containing solution. The composition of these solutions is elaborated in the Materials and Methods. (**A**) Representative *I*_K(DR)_ density evoked during 1-sec square-voltage command from −50 to +50 mV (indicated in the Inset). a: control; b: 1 μM PGG; c: 3 μM PGG; d: 10 μM PGG. (**B**) Concentration-dependent inhibition of *I*_K(DR)_ density produced by the presence of different PGG concentrations (mean ± SEM; *n* = 8). The continuous sigmoidal line over which the data points (○) are overlaid is appropriately fitted by the three-parameter logistic model (i.e., modified Hill equation). The vertical dashed line is placed at the IC_50_ value (i.e., 3.6 μM). (**C**) Time course for the PGG-induced inhibition of *I*_K(DR)_ density in a GH_3_ cell. Current density was obtained at the end of 1-sec depolarizing pulse from −50 to +50 mV. The horizontal bar shown above indicates the application of PGG (3 μM).

In an initial stage of measurements, we kept cells in Ca^2+^-free Tyrode’s solution, which contained 1 μM tetrodotoxin and 0.5 mM CdCl_2_. The reason why we used Ca^2+^-free Tyrode’s solution in these experiments is to preclude any contamination of Ca^2+^-activation in K^+^ currents that natively exist in these cells. The recording electrode was filled up with K^+^-containing internal solution. The composition of these solution is described in the Materials and Methods. Of interest, as cells were exposed to different concentrations of PGG, the *I*_K(DR)_ density elicited in response to membrane depolarization from −50 to +50 mV was progressively decreased (Figure 1A). For example, PGG at a concentration of 1 or 3 μM led to a significant reduction in the current density at the endpoint of the depolarizing pulse to 19.1 ± 2.1 pA/pF (*n* = 8, *p* < 0.05) or 8.5 ± 0.9 pA/pF (*n* = 8, *p* < 0.05), respectively, from the control level of 24.4 ± 2.2 pA/pF (*n* = 8). After washout of the agent, the current density returned to 22.6 ± 2.1 pA/pF (*n* = 7, *p* < 0.05). The concentration-dependent relation of inhibitory effect of PGG (0.3–100 μM) on the *I*_K(DR)_ density was constructed and is, hence, presented in Figure 1B. From the evaluation of the three-parameter logistic model mentioned in the Materials and Methods, the IC_50_ value of PGG required for the inhibitory effect on *I*_K(DR)_ density observed in GH_3_ cells was estimated to be 3.6 μM, and at a concentration of 100 μM, it almost fully suppress current density. Figure 1C illustrates the time course for PGG-mediated inhibition of *I*_K(DR)_ density observed in a GH_3_ cell.

### 2.2. The Effect of PGG on the Current Density Versus the Voltage Relationship of I_K(DR)_

**Figure 2:**
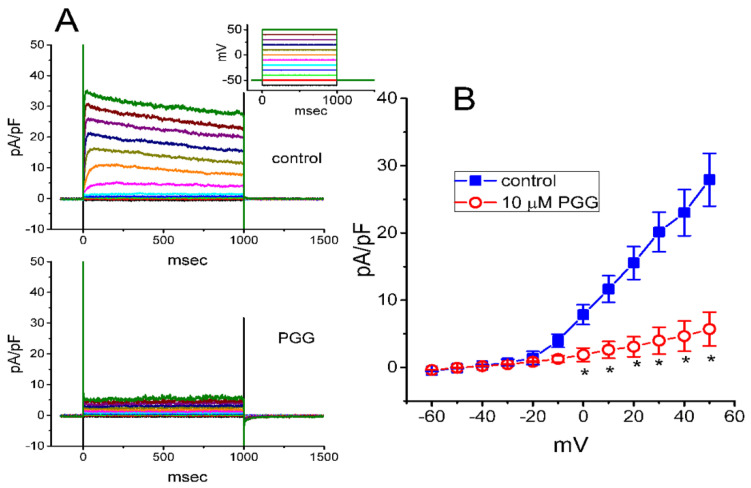
Mean current density versus voltage relationship of *I*_K(DR)_ obtained without or with the addition of PGG. The examined cells were voltage-clamped at −50 mV with different voltage commands ranging between −60 and +50 mV (representing in inset of the upper part). (**A**) Representative *I*_K(DR)_ densities achieved in the control (i.e., the absence of PGG; upper part) or during GH_3_-cell exposure to 10 μM PGG (lower part). (**B**) Mean current density versus voltage relationship of *I*_K(DR)_ in the absence (■) and presence (□) of 10 μM PGG (mean ± SEM; *n* = 7). Current density was measured at the end of each step command. The statistical analyses were made by ANOVA-2 for repeated measures, *p* (factor 1) < 0.05, *p* (factor 2) < 0.05, *p* (interaction) < 0.05, followed by post-hoc Fisher’s least-significant difference test, *p* < 0.05. * indicates a significant difference from controls obtained at the same level of membrane potential (*p* < 0.05).

Next, we wanted to determine the inhibitory effect of PGG on the current at different levels of membrane potential. As illustrated in Figure 2A,B, the current density versus the voltage relationship obtained with or without application of PGG (10 μM) is illustrated. The results disclosed that the presence of this compound lessened the *I*_K(DR)_ density measured throughout the entire voltage-clamp steps applied. That is, the whole-cell conductance of *I*_K(DR)_ density measured at the voltage ranging between 0 and +50 mV was noticed to lessen significantly from a control value of 0.39 ± 0.02 nS/pF (*n* = 7) to 0.074 ± 0.004 nS/pF (*n* = 7, *p* < 0.01). After the compound was washed out, the conductance was returned to 0.35 ± 0.02 nS/pF (*n* = 7, *p* < 0.05).

### 2.3. Effect of PGG on Erg-Mediated K^+^ Current (I_K(erg)_) in GH_3_ Cells

**Figure 3:**
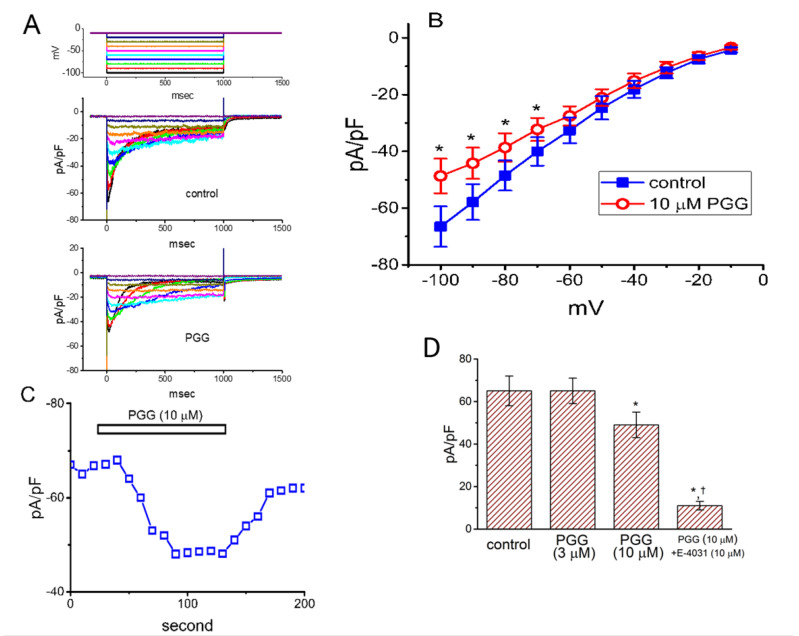
Inhibitory effect of PGG on *erg*-mediated K^+^ current (*I*_K(erg)_) identified in GH_3_ cells. In these whole-cell recordings, we bathed cells in a high-K^+^, Ca^2+^-free solution, and the pipette was filled up with K^+^-containing solution. (**A**) Representative *I*_K(erg)_ density obtained in the control (i.e., in the absence of PGG; upper) or during the exposure to 10 μM PGG (lower). The uppermost part denotes the voltage protocols applied. (**B**) Mean *I*_K(erg)_ density versus voltage relationships obtained in the absence (■) or presence (○) of 10 μM PGG (mean ± SEM; *n* = 8 for each point). Current density was measured at the start of each voltage step. The statistical analyses were made by ANOVA-2 for repeated measures, *p* (factor 1) < 0.05, *p* (factor 2) < 0.05, *p* (interaction) < 0.05, followed by post-hoc Fisher’s least-significant difference test, *p* < 0.05. * indicates a significant difference from controls measured at the same level of the hyperpolarizing step (*p* < 0.05). (**C**) Time course of *I*_K(erg)_ density obtained during cell exposure to 10 μM PGG. Each point (□) was measured at the start of a 1-sec hyperpolarizing pulse from −10 to −100 mV, and the horizontal bar shown above indicates the application of PGG (10 μM). (**D**) Summary bar graph demonstrating the effects of PGG (3 and 10 μM) and 10 μM PGG plus 10 μM E-4031 on *I*_K(erg)_ density (mean ± SEM; *n* = 7). Current density was measured at the beginning of the hyperpolarizing step from −10 to −100 mV. The statistical analyses were done by ANOVA-1, *p* < 0.05, followed by a post-hoc Fisher’s least-significant difference test, *p* < 0.05. * indicates a significant difference from control (*p* < 0.05), and ^†^ indicates a significant difference from the PGG (10 μM)-alone group (*p* < 0.05).

We further studied whether PGG was able to alter another type of voltage-gated K^+^ current (i.e., *I*_K(erg)_) present in these cells. In these whole-cell measurements, we bathed cells in high-K^+^, Ca^2+^-free solution, and the electrode was filled up with K^+^-containing solution. The examined cell was maintained at −10 mV and a series of voltage steps ranging between −100 and −10 mV were delivered to evoke deactivating *I*_K(erg)_ [18,28]. Any effects on *I*_K(erg)_ density did not occur, as the cells were exposed to PGG (3 μM). For example, at the level of −100 mV, *I*_K(erg)_ density in the control (65 ± 7 pA/pF) did not differ from that in the presence of 3 μM PGG (65 ± 6 pA/pF, *n* = 7, *p* > 0.05). However, the compound at a concentration of 10 μM significantly decreased *I*_K(erg)_ density (Figure 3A,B). We constructed the mean current density versus voltage relation of *I*_K(erg)_ achieved in its absence or presence and the results are, hence, illustrated in Figure 3B. For example, as cells were exposed to 10 μM, PGG led to a mild reduction in the *I*_K(erg)_ density at −100 mV from 66.5 ± 7.1 to 48.7 ± 6.1 pA/pF (*n* = 8, *p* < 0.05). Additionally, in the continued presence of 10 μM PGG, the subsequent addition of 10 μM E-4031, a blocker of *I*_K(erg)_, further decreased the current density to 11.2 ± 2.3 pA/pF (*n* = 8, *p* < 0.05). Figure 3C shows the time course of PGG-mediated decrease in *I*_K(erg)_ density measured from GH_3_ cells. Figure 3D illustrates the summary bar graph demonstrating the effect of PGG and PGG plus E-4031 on *I*_K(erg)_ density. As such, it is noticeable that, as compared with its effect on *I*_K(DR)_, the *I*_K(erg)_ density in GH_3_ cells is relatively less vulnerable to inhibition by adding PGG. *I*_K(erg)_ block caused by PGG might not play a role in its actions on the behavior of electrically excitable cells.

### 2.4. Biophysical Properties of Proton-Activated Cl^−^ Current (I_PAC_) Identified in GH_3_ Cells

**Figure 4:**
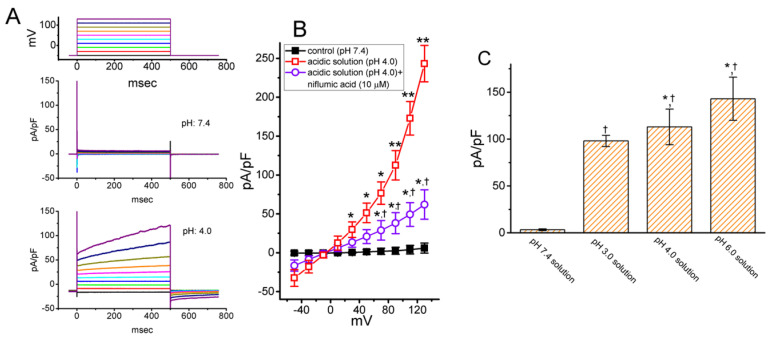
Biophysical and pharmacological properties of the proton-induced Cl^−^ current (*I*_PAC_) density identified in GH_3_ cells. The experiments were undertaken in cells which were kept bathed in Ca^2+^-free Tyrode’s solution, and the recording electrode was filled up with Cs^+^-containing solution. (**A**) Representative *I*_PAC_ densities obtained in cells exposed to the control condition (i.e., pH 7.4, upper) or to the acidic solution (i.e., pH 4.0, lower). The voltage protocol delivered is illustrated in the uppermost part. (**B**) Mean current density versus voltage relationships of *I*_PAC_ (mean ± SEM; *n* = 8 for each point). ■: control (pH 7.4); □: in the presence of acidic solution (pH 4.0); ○: in the presence of acidic solutions plus 10 μM niflumic acid. The statistical analyses were made by ANOVA-2 for repeated measures, *p* (factor 1) < 0.05, *p* (factor 2) < 0.05, *p* (interaction) < 0.05, followed by post-hoc Fisher’s least-significant difference test, *p* < 0.05. * and ** indicate a significant difference from the controls (i.e., pH 7.4) (*p* < 0.05 or *p* < 0.01, respectively) measured at the same level of the membrane potential, while ^†^ indicates a significant difference from the acidic solution (i.e., pH 4.0) (*p* < 0.05). (**C**) Summary bar graph showing the effect of acidic extracellular solution on the *I*_PAC_ density (mean ± SEM; *n* = 8 for each bar). In these experiments, the *I*_PAC_ density was taken at the end of 500-msec depolarizing step from −50 to +90 mV. The statistical analyses were done by ANOVA-1, *p* < 0.05, followed by post-hoc Fisher’s least-significant difference test, *p* < 0.05. * indicates a significant difference from the pH 3.0 solution (*p* < 0.05), and ^†^ indicates a significant difference from the pH-7.4 solution (*p* < 0.01).

The current studies have reported the presence of *I*_PAC_ emerging from different cell types [9,25,27,29]. The magnitude of these currents was also disclosed to be linked to acid-induced cell death identified in cortical neurons [27]. For these reasons, we intended to investigate whether GH_3_ cells could be functionally expressed in this type of ionic current. As the pH of extracellular media was lowered to 4.0, when the GH_3_-cell was voltage-clamped at −50 mV, the voltage steps (from −50 to +130 mV with 20-mV increments) were noted to elicit a family of large outwardly-rectifying currents, which displayed the slowly activating and deactivating time course of the current (Figure 4A,B). In other words, distinguishable from volume-sensitive Cl^−^ current [25,30,31,32], the currents tended to increase over time during the depolarizing voltage step (i.e., activation time course) and to decay slowly over time following a return to the holding potential (i.e., deactivation time course). The direction of this type of ionic current was reversed at −2 ± 2 mV (*n* = 9). When external Cl^−^ concentration was reduced to 30 or 5 mM, the value of reversal potential was changed to 62 ± 4 and 81 ± 5 mV, respectively (*n* = 6). This type of ionic currents has been identified as unraveled *I*_PAC_ recently, which was rather small when the extracellular pH was 7.4 [25,26,27]. Figure 4B shows the mean current density versus voltage relations of *I*_PAC_ measured at the end of voltage pulses as cells were exposed to Ca^2+^-free Tyrode’s solution with pH 7.4 or pH 4.0, and during the exposure to 10 μM niflumic acid, but still with the continual presence of acidic Tyrode’s solution. Therefore, a decrease in the extracellular pH value from 7.4 to 4.0 substantially led to an enhancement in the density of *I*_PAC_ elicited by rapid membrane depolarizations. As cells were depolarizing from −50 to +120 mV, the activating or deactivating time constant of *I*_PAC_ density was estimated to be 354 ± 19 or 197 ± 12 msec (*n* = 8). Within 1 min of exposing cells to niflumic acid (10 μM), the strength of the *I*_PAC_ density was strikingly reduced through the entire voltage clamp step (Figure 4B). Niflumic acid was recently reported to suppress *I*_PAC_ [26]. Figure 4C illustrates the effect of different acidic solution on the *I*_PAC_ density taken at the end of depolarizing pulse from −50 to +90 mV with a duration of 500 msec.

### 2.5. Concentration-Dependent Inhibition of I_PAC_ Density Produced by PGG and Comparisons of PGG, Chlorotoxin, Hesperetin, Kaempferol, Morin, Hydroxychloroquine (HCQ), 4-[(2-butyl-6,7-dichloro-2-cyclopentyl-2,3-dihydro-1-oxo-1H-inden-5yl)oxy] Butanoic Acid (DCPIB) and Iberiotoxin on the Current Density

**Figure 5:**
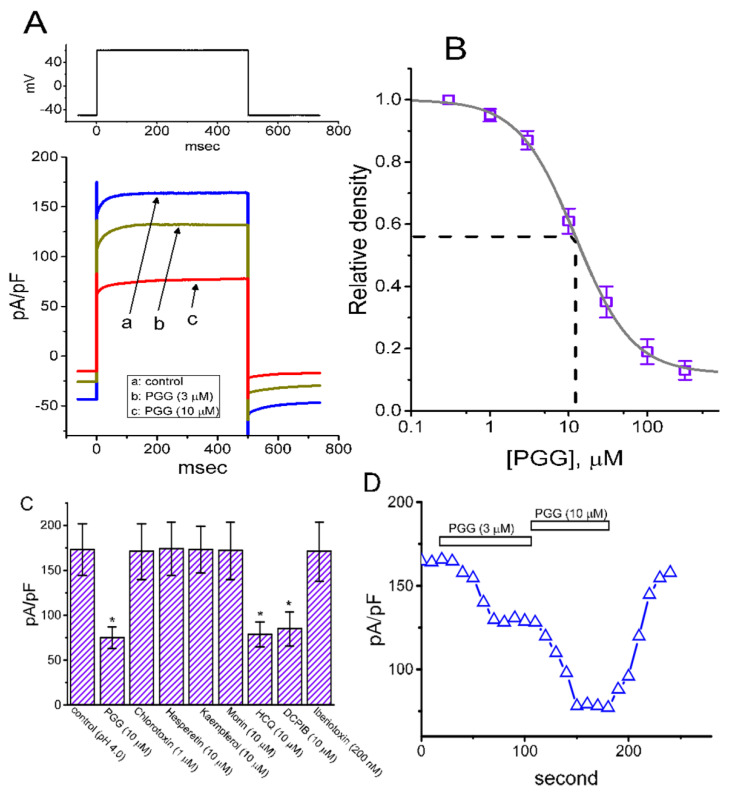
Concentration-dependent inhibitory effect of PGG on *I*_PAC_ density (**A**,**B**) and comparisons in effects of PGG, chlorotoxin, hesperetin, kaempferol, morin, hydroxychloroquine and iberiotoxin on *I*_PAC_ density (**C**). In these experiments, cells were kept immersed in acidic (i.e., pH 4.0) and Ca^2+^-free Tyrode’s solution, and the electrode was filled up with Cs^+^-containing solution. (**A**) Representative *I*_PAC_ density taken in the control (i.e., in the absence of PGG, but still exposed to acidic extracellular solution with pH 4.0) and during cell exposure to 3 μM PGG (b) or 10 μM PGG (c), but still in the presence of acidic solution. (**B**) Concentration–response relationship for the PGG-induced inhibition of *I*_PAC_ density measured at the end of the 500-msec square potential command to +50 mV from a holding potential of −50 mV (mean ± SEM; *n* = 8–9 for each point). A smooth sigmoidal line is drawn according to the three-parameter logistic model (i.e., modified Hill equation) as described in the Materials and Methods. A vertical dashed line indicates the IC_50_ value of this compound needed for its inhibition of *I*_PAC_ density. (**C**) Comparison between the effect of PGG and those of chlorotoxin, hesperetin, kaempferol, morin, hydroxychloroquine (HCQ), 4-[(2-butyl-6,7-dichloro-2-cyclopentyl-2,3-dihydro-1-oxo-1H-inden-5yl)oxy] butanoic acid (DCPIB) and iberiotoxin (mean ± SEM; *n* = 7 for each bar). The examined cell was held at −50 mV, and the command voltage pulses to +50 mV were applied. Current density was measured at the end of each depolarizing voltage-clamp pulse. The statistical analyses were done by ANOVA-1, *p* < 0.05, followed by post-hoc Fisher’s least-significant difference test, *p* < 0.05. * indicates a significant difference from control (i.e., in the presence of extracellular acid condition with pH 4.0) (*p* < 0.05). (**D**) Time course of the *I*_PAC_ density decreased by PGG (3 and 10 μM). In these recordings, cells were exposed to acidic extracellular solution with pH 4.0. Each point (△) was obtained at the end of the 500-msec depolarizing pulse from −50 to +50 mV. The horizontal bar indicates the application of 3 or 10 μM PGG.

In the next set of experiments, we made an effort to study whether the presence of PGG could exert any perturbations on *I*_PAC_ in GH_3_ cells. We allowed cells to be kept in acidic, Ca^2+^-free Tyrode’s solution with a pH value of 4.0, and the recording electrode was filled up with Cs^+^-containing solution. Figure 5A,B disclosed that the addition of PGG (0.3–300 μM) produced a concentration-dependent decrease of the *I*_PAC_ density activated by membrane depolarization. For example, in the continued presence of extracellular acidification (pH 4.0), PGG at a concentration of 3 or 10 μM lessened *I*_PAC_ density from a control value of 173 ± 29 pA/pF (*n* = 7) to 131 ± 21 pA/pF (*n* = 7, *p* < 0.05) or 75 ± 12 pA/pF (*n* = 7, *p* < 0.05), respectively. The addition of this compound was also observed to retard the activation or deactivation time course of the *I*_PAC_ density in response to membrane depolarization. The IC_50_ value needed for the inhibitory effect of PGG on *I*_PAC_ density was approximately estimated to be 12.2 μM; moreover, at a concentration of 300 μM, it almost abolished the current density. Results from these observations reflect that this compound has a depressant effect on the *I*_PAC_ density in a concentration-dependent fashion in GH_3_ cells.

The effects of PGG, chlorotoxin, hesperetin, kaempferol, morin, HCQ, DCPIB and iberiotoxin on the *I*_PAC_ density in GH_3_ cells were further examined and compared. As depicted in Figure 5C, HCQ (10 μM), similar to PGG, led to a reduction of *I*_PAC_ density. HCQ, an antimalarial and antiviral agent, has been reported to be an alkalinizing lysosomatropic drug which can accumulate in lysosomes, where it suppresses some important functions [33,34]. However, neither chlorotoxin (1 μM), hesperetin (10 μM), kaempferol (10 μM), morin (10 μM) nor iberiotoxin (200 nM) caused any striking perturbations on *I*_PAC_ density. These observations inspired us to suggest that the perturbating effect of PGG on *I*_PAC_ in GH_3_ cells be distinct from those of other polyphenols (e.g., hesperetin, kaempferol and morin). Moreover, neither chlororotixn, a nonspecific blocker of Cl^−^ channels, nor iberiotoxin, an inhibitor of large-conductance Ca^2+^-activated K^+^ channels, were noticed to have any effects on *I*_PAC_ density; however, the presence of DCPIB (10 μM), another blocker of Cl^−^ channels, could decrease the *I*_PAC_ density by 51%. Figure 5D shows the time course of the *I*_PAC_ density during exposure to 3 and 10 μM PAC.

### 2.6. Effect of PGG on Voltage-Dependent Hysteresis of I_PAC_ Elicited by Long Isosceles-Triangular Ramp Pulse

**Figure 6:**
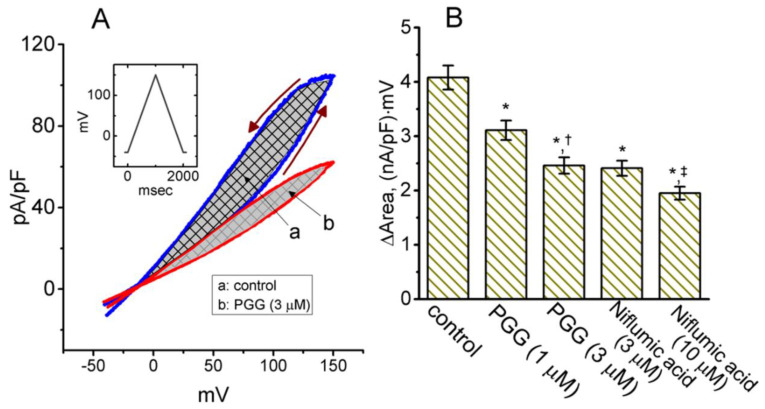
Effect of PGG on the voltage-dependent hysteresis of *I*_PAC_ density identified from GH_3_ cells. Cells were bathed in acidic (pH = 7.4), Ca^2+^-free Tyrode’s solution. (**A**) Representative *I*_PAC_ density in the absence (a) or presence (b) of 3 μM PGG. Current densities were elicited in response to 2-sec long isosceles-triangular ramp voltage command (indicated in the Inset). The arrows denote the direction of *I*_PAC_ density in which time passes. (**B**) Summary bar graph showing the effect of PGG (1 or 3 μM) or niflumic acid (3 or 10 μM) on the Δarea (indicated in the shaded area of (**A**)) of the voltage hysteresis (mean ± SEM; *n* = 8 for each bar). The statistical analyses were done by ANOVA-1, *p* < 0.05, followed by a post-hoc Fisher’s least-significant difference test, *p* < 0.05. * indicates a significant difference from control (*p* < 0.05), ^†^ indicates significant difference from the PGG (1 μM)-alone group (*p* < 0.05), and ^‡^ indicates a significant difference from the niflumic acid (3 μM)-alone group (*p* < 0.05).

The voltage hysteresis of ionic currents (i.e., a lag in the current density as the linear voltage command is changed in the opposition direction) has been disclosed with a distinct impact on the electrical behavior of action potential firing in excitable cells [18,35,36,37]. An effort was, therefore, made to evaluate whether a possible voltage-dependent hysteresis would happen in the *I*_PAC_ density measured from GH_3_ cells, and how the addition of PGG is able to modify the strength of such hysteresis. In this set of experiments undertaken in cells which were bathed in acidic (pH 4.0) and Ca^2+^-free Tyrode’s solution, as soon as the whole-cell configuration was firmly achieved, a long-lasting isosceles-triangular ramp pulse with a duration of 2 s (i.e., a ramp slope of ±0.19 V/s) was specifically designed and, through digital-to-analog conversion, then delivered to the voltage-clamped cell at a rate of 0.05 Hz. Of particular interest, as depicted in Figure 6A, the trajectories of *I*_PAC_ activated by the upsloping (i.e., the voltage change from −40 to +150 mV) ramp pulse and downsloping (i.e., the change from +150 to −40 mV) as a function of time were overly distinguishable between these two limbs. That is, the *I*_PAC_ density evoked by the upsloping (forward) limb of the isosceles-triangular voltage ramp was noticed to be lower than that in response to the downsloping (backward) limb. These observations indicated that there was a voltage-dependent hysteresis for this type of current density that inherently exists in these cells. As the ramp speed was lessened (i.e., the steepness is decreased), the hysteretic strength for *I*_PAC_ was found to become progressively elevated. The strength of the voltage hysteresis was further quantified according to the difference in the area under the curve in the forward (upsloping) and reverse (downsloping) direction, as indicated by the arrows in Figure 6A. Figure 6B illustrates a summary of the area under the curve (i.e., Δarea indicated in the shaded area) between the forward and backward current densities generated in response to a 2-sec isosceles-triangular ramp pulse. For example, as the whole-cell *I*_PAC_ was established, the addition of 1 or 3 μM PGG reduced the area up to 24 or 39%, respectively, while cell exposure to niflumic acid (10 μM), an inhibitor of *I*_PAC_, could also reduce the hysteretic area by 5%. 

### 2.7. Ineffectiveness of PGG in Perturbing Hyperpolarization-Activated Cation Current (I_h_) in GH_3_ Cells

**Figure 7:**
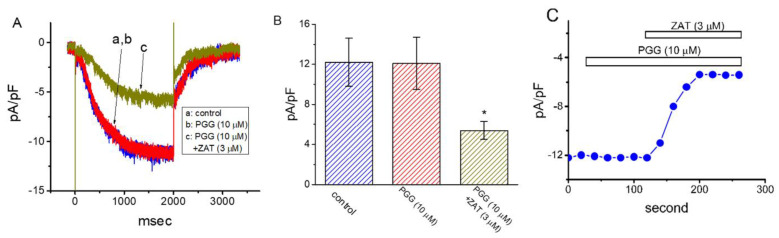
Inability of PGG to perturb the hyperpolarization-activated cation current (*I*_h_) identified from GH_3_ cells. In this set of experiments, cells were bathed in a Ca^2+^-free Tyrode’s solution that contained 1 μM tetrodotoxin, and the electrode was filled up with K^+^-containing solution. (**A**) Representative *I*_h_ densities obtained in the control, and during the exposure to 10 μM PGG or 10 μM PGG plus 3 μM zatebradine (ZAT). In the experiments on PGG plus zatebradine, as the cells were continually exposed to PGG, further addition of zatebradine was applied. (**B**) Summary bar graph showing the effects of PGG and PGG plus zatebradine (ZAT) on the density of hyperpolarization-evoked *I*_h_ (mean ± SEM; *n* = 7 for each bar). Each current density in (**A**,**B**) was taken at the end of 2-sec hyperpolarizing step to −120 mV from a holding potential of −40 mV. The statistical analyses were done by ANOVA-1, *p* < 0.05, followed by post-hoc Fisher’s least-significant difference test, *p* < 0.05. * indicates a significant difference from control (*p* < 0.05). (**C**) Time course of effect of PGG (10 μM) and PGG (10 μM) plus ZAT (3 μM) on *I*_h_ density. *I*_h_ density was taken at the end of 2-sec hyperpolarizing step from −40 to −120 mV.

It was additionally evaluated whether the presence of PGG affected any modifications on different types of ionic current (e.g., *I*_h_) found in these cells [18,28]. To measure *I*_h_, GH_3_ cells were bathed in Ca^2+^-free Tyrode’s solution, and the recording electrode was filled up with K^+^-containing solution. The examined cell was voltage-clamped at −40 mV and sustained hyperpolarization to −120 mV was applied to evoke *I*_h_ density [37,38]. As depicted in Figure 7, PGG at a concentration of 10 μM alone did not cause any effect on *I*_h_ responding to maintained hyperpolarization applied from −40 to −120 mV. However, as PGG was continually present, further addition of zatebradine (3 μM) noticeably reduced *I*_h_ density in combination with a conceivable slowing in activating time course of the current. Zatebradine was previously reported to suppress *I*_h_ amplitude effectively [38]. Hence, distinguishable from either *I*_K(DR)_ or *I*_PAC_ detected above, the *I*_h_ in these cells failed to be influenced by adding PGG.

### 2.8. Inhibitory Effect of PGG on Voltage-Gated Na^+^ Current (I_Na_) in GH_3_ Cells

**Figure 8:**
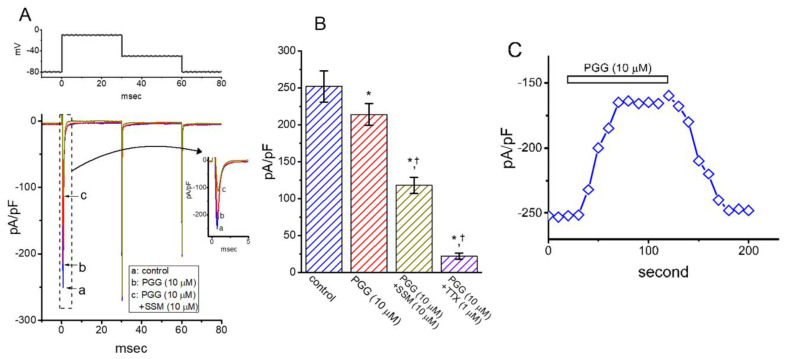
Inhibitory effect of PGG on the density of voltage-gated Na^+^ current (*I*_Na_) in GH_3_ cells. The experiments were conducted in cells kept to be bathed in Ca^2+^-free Tyrode’s solution containing 10 mM tetraethylammonium chloride and 0.5 mM CdCl_2_, and we filled up the recording electrode by using Cs^+^-containing solution. (**A**) Representative *I*_Na_ densities obtained in the control (a) and during the exposure to 10 μM PGG (b) or 10 μM PGG plus 10 μM sesamin (SSM) (c). The voltage protocol delivered is presented in the upper part, while inset indicates an expanded record from the dashed box. Of note, the voltage-clamp profile used in the present study was applied to measure transient and deactivating densities of *I*_Na_. (**B**) Summary bar graph revealing effects of PGG, PGG plus sesamin (SSM), and PGG plus tetrodotoxin (TTX) on the density of depolarization-evoked *I*_Na_ (mean ± SEM; *n* = 8). The density of peak *I*_Na_ was taken at the start of brief depolarization from −80 to −10 mV. The statistical analyses were done by ANOVA-1, *p* < 0.05, followed by post-hoc Fisher’s least-significant difference test, *p* < 0.05. * indicates a significant difference from control (*p* < 0.05), and ^†^ significantly different from PGG (10 μM) alone group (*p* < 0.05). (**C**) Time course showing the effect of PGG (10 μM) on peak *I*_Na_ density. Each current density (◇) was taken at the beginning of 30-msec depolarizing pulse from −80 to −10 mV.

Epicatechin-3-gallate, a flavonoid polyphenol extracted from green tea [24], has been previously demonstrated to modify the amplitude and gating kinetics of INa identified in cultured neonatal ventricular myocytes [39]. Therefore, we continued to test whether the presence of PGG has any modifications on *I*_Na_ in GH_3_ cells. In this stage of experiments, cells were kept to be bathed in Ca^2+^-free Tyrode’s solution, which contained 10 mM tetraethylammonium chloride and 0.5 mM CdCl_2_, while a recording electrode was filled up with Cs^+^-containing solution, the composition of which was stated in Materials and Methods. As revealed from the results of Figure 8A,B, PGG at a concentration of 10 μM could mildly but significantly inhibit peak *I*_Na_ density (measured at the beginning of depolarizing pulse) elicited by rapid membrane depolarization. For example, as the examined cell was depolarized from −80 to −10 mV, addition of 10 μM PGG lessened peak *I*_Na_ density from 252 ± 21 to 214 ± 15 pA/pF (*n* = 8, *p* < 0.05). After washout of the compound, the current density returned to 248 ± 18 pA/pF (*n* = 7, *p* < 0.05). Figure 8C depicts the time course of the PGG-induced decrease of the peak *I*_Na_ density. However, in contrast to the action of the epicatechin-3-gallate on the amplitude and gating of peak *I*_Na_ previously stated [39], the time course of neither activation, inactivation nor deactivation for depolarization-elicited *I*_Na_ was clearly perturbed by adding PGG. By extension, the subsequent application of either 10 μM sesamin (SSM) or 1 μM tetrodotoxin, still in the continued presence of 10 μM PGG, was effective at suppressing the peak density of *I*_Na_ (Figure 8B). Sesamin, a furofuran lignan of sesame oil, was recently reported to decrease *I*_Na_ in response to brief depolarization [22]. In keeping with previous observations, cell exposure to sesamin led to a decrease in peak *I*_Na_ density, accompanied by a marked increase in the inactivation or deactivation time curse of the *I*_Na_ density [22].

### 2.9. Inhibitory Effect of PGG on Ramp-Induced I_K(DR)_ Identified in Human Cardiac Myocytes (HCM)

**Figure 9:**
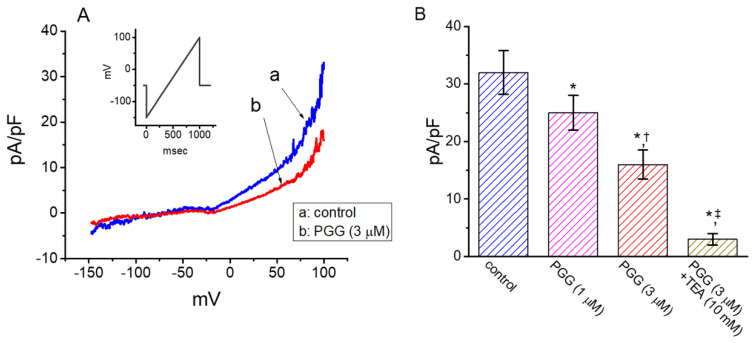
Inhibitory effectiveness of PGG in the density of ramp voltage-induced *I*_K(DR)_ recorded from human cardiac myocytes (HCM). In this set of whole-cell current recordings, we kept HCM in Ca^2+^-free Tyrode’s solution and then filled up the electrode by using K^+^-enriched solution. (**A**) Ramp-induced *I*_K(DR)_ density obtained in the absence (a) or presence of 3 μM PGG (b). The inset indicates the linear ramp protocol delivered to the cell. (**B**) Summary bar graph showing effect of PGG and PGG plus tetraethylammonium chloride (TEA) on *I*_K(DR)_ density in HCM (mean ± SEM; *n* = 8 for each bar). Current density was measured at the level of +100 mV. The statistical analyses were done by ANOVA-1, *p* < 0.05, followed by post-hoc Fisher’s least-significant difference test, *p* < 0.05. * indicates a significant difference from control (*p* < 0.05), ^†^ indicates a significant difference from the PGG (1 μM)-alone group (*p* < 0.05), and ^‡^ indicates a significant difference from the PGG (3 μM)-alone group (*p* < 0.05).

PGG was previously reported to exert beneficial effects in tissue mechanics, such as heart valves [13,14]. Thus, the possible perturbations of PGG on *I*_K(DR)_ in HCM were further evaluated. The measurements were undertaken in HCM bathed in Ca^2+^-free Tyrode’s solution containing 1 μM tetrodotoxin and 0.5 mM CdCl_2_, and the pipette solution was enriched in high K^+^ solution. As depicted in Figure 9, when the linear ramp pulse from −150 to +100 mV with a duration of 1 s was applied to evoke *I*_K(DR)_ density, cell exposure to 3 μM PGG resulted in an evident reduction in *I*_K(DR)_ density. For example, at the level of +100 mV, the presence of 3 μM PGG decreased the current density from 31.9 ± 3.8 to 16.1 ± 2.5 pA/pF (*n* = 8, *p* < 0.05). Subsequent application of 10 mM tetraethylammonium chloride, still in the presence of PGG, was noticed to decrease the current density further to 2.9 ± 1.0 pA/pF (*n* = 8, *p* < 0.05). As such, the experimental data reflect that, similar to findings stated above in GH_3_ cells and previously in regard to effects of flavonoids [24,40], this compound is effective at suppressing the *I*_K(DR)_ density existing in HCM. 

## 3. Discussion

The notable findings achieved from this study are that the presence of PGG, a natural polyphenol compound, has the propensity to interact with different types of plasmalemmal ion channels to modify their current densities. The IC_50_ value needed for the inhibition of the *I*_K(DR)_ or *I*_PAC_ density caused by the presence of this compound in GH_3_ cells was shown to be 3.6 or 12.2 μM, respectively, the value of which was within the pharmacologically applied concentrations reported previously [4,26,41]. For example, it has been previously demonstrated that after 8 days of treatment, PGG decreased the level of extracellular hepatitis B virus in a concentration-dependent manner with an IC_50_ of 1.1 μM and also decreased the HBeAg level by 25% at a concentration of 4.2 μM [42]. However, its presence mildly suppressed the *I*_K(erg)_ or *I*_Na_ density as well as failed to alter the *I*_h_ density. The *I*_K(DR)_ density in HCM was also subject to be suppressed by this compound.

In the current study, we presented the expression of *I*_PAC_ in GH_3_ cells, the biophysical and pharmacological properties of which tend to be in accordance with those described previously [9,29]. More importantly, the voltage-dependent hysteresis of *I*_PAC_ density in response to the long isosceles-triangular ramp pulse was further demonstrated in these cells, although the hysteresis in the decay between the Ca^2+^-activated Cl^−^ current and intracellular Ca^2+^ has been previously reported [43]. The strength of such voltage-dependent hysteresis might serve a role in influencing the electrical behavior of excitable cells such as GH_3_ cells [18]. In keeping with previous observations made in different types of ionic currents [35,36,37], the *I*_PAC_ density in GH_3_ cells was noted to undergo hysteretic perturbation. Therefore, either its voltage dependence or a significant mode shift, where there is a voltage sensitivity in the gating charge movements of the current, might potentially appear. In other words, the strength of the *I*_PAC_ density could be contingent on the pre-existing state or conformation of the PAC channel. In this study, we also evaluated the possible modifications of PGG on such a non-equilibrium property of *I*_PAC_ density present in GH_3_ cells. The present results showcased that the presence of this compound was allowed to diminish the hysteretic strength (i.e., Δarea indicated in the shaded area of Figure 6B) involved in the voltage dependent activation of *I*_PAC_ density. However, it needs to be mentioned that in data such as quantitative polymerase chain reaction, immunohistochemistry and western blotting the presence of *I*_PAC_ expressed in GH_3_ cells should be confirmed. How the profile of different Cl^−^ channel-blockers profile influences *I*_PAC_ also needs to be further established, although niflumic acid or DCPIB could suppress it.

PGG may affect protein receptor conformation by interacting closely with the membrane bilayer surrounding their assembly, owing to its particular mode of insertion specifically involving the galloyl moieties B and E of PGG over the membrane surface, leaving the other residues in close contact with the aqueous medium. The stacking interaction between PGG and the components of the surface of the phospholipid bilayer, enhancing the stability of this layer [44]. It enables us to speculate that during cell exposure, the PGG molecule could favor the covalently linked bond formation, leading possibly to the PGG-ion channel conjugates. Of notice, the inhibition by PGG or HCQ of *I*_PAC_ density and gating may additionally confer its effectiveness on different cellular functions (e.g., autophagic or microbicidal activity) in different cell types including pituitary adenomatous cells [45]. Whether similar findings with respect to the PAC-channel presence and inhibitory effect of PGG or HCQ on *I*_PAC_ happen in other types of native cells occurring in vivo remains to be further resolved.

A current paper showed that, in pancreatic acinar cells, the Cl^−^ channels induced by extracellular acidification could be dependent on the production of reactive oxygen [46]. However, as cells were continually exposed to extracellular acidic milieu with pH 4.0, the subsequent addition of dithiothreitol (10 μM), a sulfhydryl reducing agent, did not suppress the strength of *I*_PAC_ density. Therefore, the *I*_PAC_ found in GH_3_ cells could be unrelated to the production of reactive oxygen species; however, it indeed was sensitive to being blocked by adding niflumic acid, HCQ, DCPIB or PGG. In reality, it seems unlikely that PGG-mediated inhibition of *I*_K(DR)_ or *I*_PAC_ observed in the current study is mediated mainly through its anti-oxidative activities [4,15]. 

Similar to PGG, some flavonoids (e.g., hesperetin, kaempferol or morin) are embraced to be biologically active polyphenolic compounds. However, in our study, none of these compounds revealed any inhibitory effects on *I*_PAC_ density, whereas the addition of PGG was able to exert a concentration-dependent inhibitory effect on the current density. Findings from these observations allow us to suggest, therefore, that the mechanisms through which PGG suppressed *I*_PAC_ could not be solely explained by multiple hydroxyl groups existing in the molecule, although from its structure-activity relationship, the appearance of specific hydroxyl groups gives it a high bioactivity [44]. However, as GH_3_ cells were exposed to HCQ, the density of *I*_PAC_ also noticeably decreased. As acidification of the vacuole (i.e., phagocytic vacuole) could reach pH 4.0 [47], it would be worthwhile to determine whether the magnitude of *I*_PAC_ in lysosomal membrane perturbed by the presence of HCQ [33,34] participates in drastic changes in the activities of degradative enzymes inside lysosomes or other organelles [48]. Indeed, luminal pH in the organelles has been previously demonstrated to be crucial to their function, e.g., post-translational modifications, along the secretory pathway, ligand trafficking in endosomes and macromolecule degradation in lysosomes are all highly dependent on pH [47].

Nonetheless, from a pharmacological standpoint, PGG-perturbed adjustment of ionic currents, including *I*_K(DR)_ and *I*_PAC_, might contribute to its effectiveness in altering functional activities of endocrine or neuroendocrine cells appearing in vivo [11,12,41]. To what extent PGG or tannic acid affects the anti-viral activity (e.g., inhibiting the entry of viral particles), as reported previously [6,8,10], and is mediated through its perturbations on plasmalemmal ion channels merits re-evaluation. 

## 4. Materials and Methods

### 4.1. Chemicals, Drugs and Solutions Used in the Current Study

Pentagalloylglucose (PGG, penta-*O*-galloyl-β-d-glucose; 1,2,3,4,6-pentagalloyl glucose, [(2*R*,3*R*,4*S*,5*R*,6*S*)-3,4,5,6-tetrakis[(3,4,5-trihydroxybezoyl)oxy]oxan-2yl]methyl 3,4,5-trihydroxybenzoate, C_41_H_32_O_26_, https://pubchem.ncbi.nlm.nih.gov/compound/Pentagalloylglucose (PubChem CID: 65238)) was acquired from MedChemExpress (Bio-genesis Technologies, Taipei, Taiwan). 4-[(2-Butyl-6,7-dichloro-2-cyclopentyl-2,3-dihydro-1-oxo-1H-inden-5yl)oxy] butanoic acid (DCPIB), dithiothreitol, E-4031, hydroxychloroquine, niflumic acid, tetraethylammonium chloride (TEA) and tetrodotoxin were acquired from Sigma-Aldrich (Merck Ltd., Tainan, Taiwan), iberiotoxin was from Alomone (Asia Bioscience, Taipei, Taiwan) and hesperetin, kaempferol and morin were from Wuhan ChemFaces Biochemical Co., Ltd. (Asia Bioscience, Taipei, Taiwan). Sesamin was kindly provided by Dr. Ping-Chung Kuo (School of Pharmacy, National Cheng Kung University Medical College, Tainan, Taiwan), while chlorotoxin was a gift acquired from Professor Dr. Woei-Jer Chuang (Department of Biochemistry, National Cheng Kung University Medical College, Tainan, Taiwan). Unless otherwise stated, culture media (e.g., Ham’s F-12 medium), fetal bovine serum, horse serum, L-glutamine and trypsin/EDTA were acquired from HyClone^TM^ (Thermo Fisher; Level Biotech, Tainan, Taiwan), while other chemicals, such as CdCl_2_, CsOH, CsCL, EGTA and HEPES, were of analytical grade. In this study, we obtained the reagent water from a Milli-Q Ultrapure Water Purification System (18.2 MΩ-cm) (Merck Millipore, Taipei, Taiwan) in all experiments.

The composition of extracellular or intracellular solution (i.e., HEPES-buffered normal Tyrode’s solution) used in this work was elaborated in Table 1. As PGG can undergo an oxidation reaction, which depends on the pH, the pH in the bath solution was kept constant at 7.4; moreover, as extracellular acidification was needed, the solution was rapidly switched to acidic Tyrode’s solution (e.g., pH 4.0). For measurement of proton-activated Cl^−^ current (*I*_PAC_), the pH was adjusted to different acidic values (from 6.0 to 3.0) with an HCl-Na_3_-citrate buffer system, and the osmolarity of the solution remained unchanged. Since the low pH solution was buffered with 1 M HCl solution by using HCl-Na_3_-citrate buffer, it is possible that the Cl^−^ concentration in low-pH solution was slightly higher than those in normal-pH solution. The pipette solution and culture medium were filtered on the day of use with a sterile Acrodisc^®^ syringe filter with a 0.2-μm Supor^®^ membrane (Bio-Check; New Taipei City, Taiwan).

### 4.2. Cell Culture

The pituitary adenomatous cell line, GH_3_, was acquired from the Bioresources Collection and Research Center (BCRC-60015, http://catalog.bcrc.firdi.org.tw/BcrcContent?bid=60015; Hsinchu, Taiwan), while the human cardiac myocytes (HCM), originally isolated from the ventricles of the adult heart, were acquired from PromoCell^®^ (Biochiefdom International Co., New Taipei City, Taiwan). GH_3_ cells were grown in Ham’s F-12 medium supplemented with 2.5% (*v/v*) fetal calf serum, 15% (*v/v*) horse serum and 2 mM L-glutamine, while HCM was cultured in PromoCell Growth Medium (Biochiefdom). Cells were maintained in a humidified atmosphere of 5% CO_2_ and 95% air at 37 °C. We commonly made the subcultures by trypsinization (0.025% trypsin solution (HyClone^TM^) containing 0.01% sodium *N*,*N*-diethyldithiocarbamate and EDTA). The experiments were undertaken five or six days after cells were cultured up to 60–80% confluence.

### 4.3. Electrophysiological Measurements

On the day of the experiments, cells (e.g., GH_3_ cells or HCM) were dispersed with a 1% trypsin/EDTA solution and a few drops of cell suspension was rapidly placed in a custom-built recording chamber, which was securely mounted on the stage of an inverted DM-IL microscope (Leica; Major Instruments, Kaohsiung, Taiwan). They were allowed to be immersed at room temperature (20–25 °C) in HEPES-buffered normal Tyrode’s solution, the composition of which is detailed above. When the cells were kept to settle onto the bottom of the chamber, the measurements were performed. We measured ion currents in whole-cell mode of the patch-clamp technique with dynamic adaptive suctioning (i.e., decremental change of suction pressure responding to a progressive increase in the seal resistance), with the aid of either an Axoclamp-2B (Molecular Devices; Advance Biotech, New Taipei City, Taiwan) or an RK-400 (Biol-Logic, Claix, France) amplifier [49]. Consistent with previous observations [50], the formation of a bleb of membrane lipid in the electrode tip based on microscopic observation of giga-Ω seal formation was noticed in our study. The microelectrodes used in the recordings were drawn from Kimax-51 borosilicate unfilamented capillaries with a 1.5–1.8 mm outer diameter (#34500; Kimble’ Dogger, New Taipei City, Taiwan) by use of a two-stage vertical micropipette puller (PP-83; Narishige, Major Instruments, New Taipei City, Taiwan), and their tips were lightly heat polished with an MF-83 microforge (Narishige). The tip resistance of these electrodes was measured to range between 3 and 5 MΩ, as they were filled up with different internal solutions, as stated above. During the measurements, the recorded area on the vibration-free table was shielded by using a Faraday cage (Scitech, Seoul, Korea). Liquid junction potential developed at the pipette tip, as the composition of the pipette solution was different from that in the bath. Electronic compensation before giga-Ω seal formation would eliminate the potential. However, as the whole-cell mode was established, the amplifier used would be applied back to liquid junction potential to the cell membrane. Therefore, in the whole-cell configuration, voltage-command applied needs to be corrected. All potentials were corrected for junction potential (−13.1 ± 2 mV, *n* = 15 GH_3_ cells; −13.3 ± 2 mV, *n* = 12 HCMs).

### 4.4. Data Recordings

Recording signals comprising potential and current traces were monitored on an HM-507 oscilloscope (Hameg, East Meadow, NY, USA) and digitally stored online at 10 kHz in a Sony VAIO CS series laptop computer (VGN-CS110E; Kaohsiung, Taiwan), equipped with 1440A digitizer (Molecular Devices). During the recordings with analog-to-digital and digital-to-analog conversion, the latter device was actually controlled by pCLAMP 10.7 software (Molecular Devices) run on Microsoft Windows 10 (Redmond, WA, USA). The laptop computer used was put on the top of an adjustable Cookskin stand (Ningbo, Zhejiang, China) for efficient manipulation during the experiments. To ensure digitalization, some of recordings were digitally collected by PowerLab 2/26 acquisition system (AD Instruments; Gerin Technology Co., Tainan, Taiwan). During measurements, we carefully exchanged the solutions between different sets of recordings through a home-made gravity-driven type of bath perfusion.

### 4.5. Data Analyses

In attempts to evaluate the concentration-dependent inhibitory effects of PGG on *I*_K(DR)_ or *I*_PAC_ density, *I*_K(DR)_ and *I*_PAC_ densities were, respectively, evoked by 1-sec or 500-msec depolarizing pulse to +50 mV from the holding potential of −50 mV, and current densities taken with or without the addition of different PGG concentrations were measured at the end of depolarizing pulse. The *I*_K(DR)_ or *I*_PAC_ density in the control (i.e., PGG was not present) was taken as 100%, and those taken in different PGG concentrations were then compared. The concentration required to inhibit 50% of current density (i.e., *I*_K(DR)_ or *I*_PAC_ density) was determined according to the three-parameter logistic model (i.e., a modified form of sigmoidal Hill equation) by use of goodness-of-fit assessments:

$$\mathrm{Relative}\;\mathrm{amplitude}=\frac{\left(1-a\right)\times {\left[\mathrm{PGG}\right]}^{-n_{H}}}{{\mathrm{IC}}_{50}^{-n_{H}}+{\left[\mathrm{PGG}\right]}^{-n_{H}}}+a$$

where IC_50_ or n_H_ is the PGG concentration (i.e., [PGG]) required for a 50% inhibition or the Hill coefficient, respectively; moreover, maximal inhibition (i.e., 1 − a) was approximated from the equation.

### 4.6. Statistical Analyses

Linear or nonlinear curve fitting to experimental data sets was acquired with the least-squares minimization procedure by using different maneuvers, such as Microsoft Excel-embedded “Solver” (Microsoft) and 64-bit OriginPro^®^ program (OriginLab; Schmidt Scientific, Kaohsiung, Taiwan). The averaged results are presented as the mean ± standard error of the mean (SEM) with sample sizes (*n*) denoting the cell numbers from which the experimental data were counted. Paired or unpaired Student’s *t*-tests were initially applied for the statistical analyses. As the statistical difference among different groups was necessarily determined, we performed either analysis of variance (ANOVA)-1 or ANOVA-2 with or without repeated measures followed by post-hoc Fisher’s least-significant difference test. Statistical analyses were performed using the SPSS 20 statistical software package (AsiaAnalytics, Taipei, Taiwan). Statistical significance was determined at a *p*-value of <0.05 or <0.01 as indicated in the figure legend.

## Figures and Tables

**Table 1:** The composition of extracellular and intracellular solutions used in this study.

Solution’s Name	Milieu	Composition (in mM)
Normal Tyrode’s solution	Extracellular	136.5 NaCl, 5.4 KCl, 1.8 CaCl_2_, 0.53 MgCl_2_, 5.5 glucose and 5.5 HEPES, adjusted with NaOH to pH 7.4
Ca^2+^-free Tyrode’s solution	Extracellular	136.5 NaCl, 5.4 KCl, 0.53 MgCl_2_, 5.5 glucose and 5.5 HEPES, adjusted with NaOH to pH 7.4
TEA-containing solution *	Extracellular	136.5 NaCl, 5.4 KCl, 1.8 CaCl_2_ (or 0 CaCl_2_), 0.53 MgCl_2_, 5.5 glucose, 10 TEA and 5.5 HEPES, adjusted with NaOH to pH 7.4
High-K^+^, Ca^2+^-free solution	Extracellular	130 KCl, 10 NaCl, 3 MgCl_2_ and 5 HEPES, adjusted with KOH to pH 7.4
Acidic solution	Extracellular	136.5 NaCl, 5.4 KCl, 0.53 MgCl_2_, 5.5 glucose and 5.5 Na_3_-citrate, adjusted with HCl to pH 3.0, 4.0 or 6.0.
K^+^-aspartate solution	Intracellular	140 K-aspartate, 1 MgCl_2_, 3 Na_2_ATP, 0.1 Na_2_GTP, 0.1 EGTA and 5 HEPES, adjusted with KOH to pH 7.2
CsCl solution	Intracellular	140 CsCl 140, 1 MgCl_2_, 3 Na_2_ATP, 0.1 Na_2_GTP, 0.1 EGTA and 5 HEPES, titrated with CsOH to pH 7.2
Cs^+^-aspartate solution	Intracellular	140 Cs-aspartate, 1 MgCl_2_, 3 Na_2_ATP, 0.1 Na_2_GTP, 0.1 EGTA and 5 HEPES, adjusted with CsOH to pH 7.2

* The osmolarity of TEA-containing solution was slightly different from that in normal Tyrode’s solution.

## Abbreviations

ANOVA	Analysis of variance
DCPIB	4-[(2-butyl-6,7-dichloro-2-cyclopentyl-2,3-dihydro-1-oxo-1H-inden-5-yl)oxy] butanoic acid
HCM	Human cardiac myocytes
HCQ	Hydroxychloroquine
*I*_h_	Hyperpolarization-activated cation current
*I*_K(DR)_	delayed-rectifier K^+^ current
*I*_K(erg)_	*erg*-mediated K^+^ current
*I*_Na_	voltage-gated Na^+^ current
*I*_PAC_	proton-activated Cl^−^ current
K_V_	voltage-gated K^+^ channel
PAC channel	proton-activated Cl^−^ channel
PGG	pentagalloylglucose (penta-O-galloyl-β-D-glucose)
SEM	standard error of the mean
TEA	tetraethylammonium chloride
